# Co-existence of breast and ovarian cancers in BRCA germ-line mutation carriers

**DOI:** 10.3332/ecancer.2008.109

**Published:** 2008-11-25

**Authors:** A Dilawari, J Cangiarella, J Smith, A Huang, A Downey, F Muggia

**Affiliations:** 1 Department of Medical Oncology, NYU Langone School of Medicine, New York, NY 10016, USA; 2 Department of Pathology, NYU Langone School of Medicine, New York, NY 10016, USA; 3 Department of Nursing, NYU Clinical Cancer Institute, New York, NY 10016, USA

## Abstract

The co-existence of breast and ovarian cancers in the same individual should raise suspicion of a hereditary process. Patients with either BRCA1 or BRCA2 germ-line mutations have an average risk of 39% and 11% respectively of developing ovarian cancer by the age of 70; they have a risk of 35–85% of developing breast cancer in their lifetime. We report here unusual pathologic features in a BRCA2 germ-line mutation carrier recently diagnosed with synchronous breast and ovarian cancers, and summarize the findings in six other women who were diagnosed with ovarian cancer either simultaneously with the diagnosis of breast cancer or at varying times after the diagnosis. While in most instances this may be a coincidental occurrence in highly susceptible individuals, the patient we highlight raises the provocative hypothesis that at times breast cancer metastasizes to the ovaries of mutation carriers and stimulates the development of an ovarian cancer as well as other cancers. In addition, these ovarian cancers may have different mechanisms of metastases predisposing them to travel to unusual sites.

## Introduction

The co-existence of breast and ovarian cancers in the same individual should raise suspicion of a hereditary process. Patients with either BRCA1 or BRCA2 germ-line mutations have an average risk of 39% and 11% respectively of developing ovarian cancer by the age of 70; they have a risk of 35–85% of developing breast cancer in their lifetime. The presence of these mutations is approximately one in 400 and one in 600, respectively, in the general population; but in certain ethnic groups, such as the Ashkenazi Jewish population, these mutations are found at an increased incidence of more than 2%.

We report here unusual pathologic features in a BRCA2 germ-line mutation carrier recently diagnosed with synchronous breast and ovarian cancers and summarize the findings in six other women who were diagnosed with ovarian cancer either simultaneously with the diagnosis of breast cancer or at varying times after the diagnosis. While in most instances this may be a coincidental occurrence in highly susceptible individuals, the patient we highlight raises the provocative hypothesis that at times breast cancer metastasizes to the ovaries of mutation carriers and stimulates the development of an ovarian cancer as well as other cancers. In addition, these ovarian cancers may have different mechanisms of metastases predisposing them to travel to unusual sites.

## Patients

Table 1 provides demographic details of the patients' diagnoses, together with clinical features and BRCA germ-line mutation status. We provide the clinical and pathological features of patient 1, who was identified to have the BRCA2 mutation (after her brother as well as paternal cousins were diagnosed with breast cancer) in greater detail because of the unusual pathology observed in this synchronous presentation: the histology in the ovary is somewhat consistent with metastases from the breast, but peritoneal metastases that were identified had a distinct papillary serous morphology indicating a different primary.

### Patient 1: clinical and pathological findings

**Figure 1:**
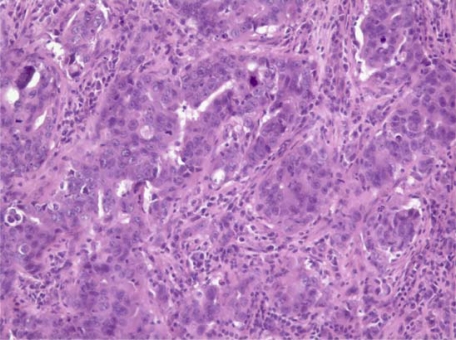
Histologic examination of the breast mass showed a high-grade carcinoma with pleomorphic nuclei and prominent nucleoli infiltrating predominantly in sheets with occasional glandular formations. A prominent lymphoplasmacytic infiltrate surrounds clusters of tumour cells. (Haematoxylin and eosin, ×200)

**Figure 2:**
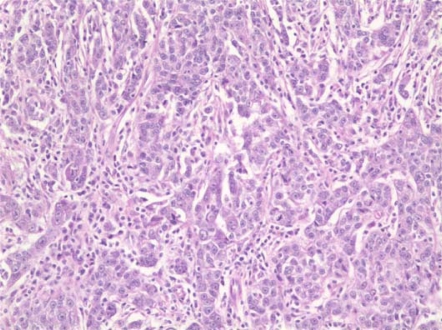
Histologic examination of the left-ovarian mass shows a histologic picture similar to the breast carcinoma with sheets of high-grade tumour cells surrounded by a lymphoplasmacytic infiltrate. Mitotic figures are easily identified. (Haematoxylin and eosin, ×200)

**Figure 3:**
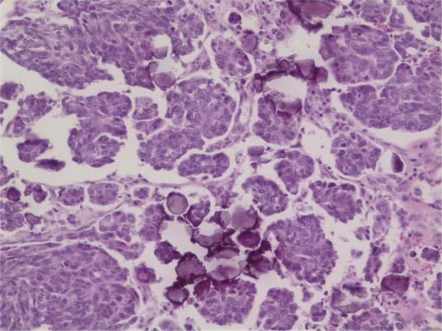
Histologic examination of the omental mass shows tumour cells arranged in papillary groupings with numerous psammoma bodies. A lymphoplasmacytic infiltrate is not seen. (Hematoxylin and eosin, ×200)

This 73-year-old woman, previously in good health, felt a mass in her left breast. Following mammographic evaluation, she had fine-needle aspiration revealing invasive ductal carcinoma. She underwent lumpectomy and sentinel node biopsy; the tumour was a 2.5-cm infiltrating ductal carcinoma with medullary features (Figure 1). Two of ten axillary nodes were involved with cancer. The oestrogen receptors (ER) and progesterone receptors (PR) were strongly positive, and the Her2 was not over-expressed. A staging PET/CT was obtained because of vague, but persistent, abdominal symptoms and 19F-deoxyglucose uptake was present in a left-adnexal mass (4.1 × 2.5 cm) and a 3.9-cm mass in the peritoneal cavity. This led to a laparoscopic resection during which frozen sections of the left tube and ovary were consistent with metastatic carcinoma from primary mammary cancer. Resection of pelvic organs and the omental peritoneal mass by the splenic flexure was deemed appropriate. Subsequent sections confirmed the left ovary to be replaced by a metastatic cancer with medullary features (Figure 2), while the omental mass had classical features of papillary serous cancer (Figure 3). The sigmoid fat tissue had involvement of metastatic adenocarcinoma of mammary origin.

The patient was treated with carboplatin + paclitaxel with normalization of the PET/CT. Since both cancers appeared to co-exist, it was decided to proceed with additional chemotherapy with carboplatin + liposomal doxorubicin, followed by maintenance liposomal doxorubicin, which she remained on for ten months without evidence of relapse. In addition, she began treatment with anastrozole, which is planned for five years (in the absence of relapse). The significance of her family history became apparent after genetic testing revealed a BRCA2 mutation.

Of note, one year later, patient complained of mild left upper quadrant abdominal pain. A PET/CT was performed that showed a 2.5-cm mass with elevated SUV around the pancreas. The patient was referred to a surgeon who performed a diagnostic laparoscopy. At that time, a small liver mass was found and was biopsied. The mass showed metastatic adenocarcinoma of pancreaticobiliary origin. The tumour had a complex glandular architecture composed of cuboidal to tall columnar cells with basophilic vacuolated cytoplasm and irregular hyperchromatic nuclei and prominent nucleoli. Numerous mitotic figures were seen. Immunohistochemical stains showed strong and diffuse positivity for CK7 and CD20 and negativity for CDX2, oestrogen receptor and WT-1.

## Pathology

Histologic examination of the lumpectomy specimen (Figure 1) shows a 1.4-cm invasive ductal adenocarcinoma with medullary features. The tumour cells were arranged in sheets with rare tubular formations surrounded by a prominent lymphoplasmacytic infiltrate. The tumour cells were pleomorphic with marked variation in size and shape, coarse chromatin and prominent nucleoli. Mitotic figures were numerous. No *in situ* carcinoma was identified. Immunohistochemical stains were positive for oestrogen and progesterone receptors and negative for her-2-neu.

A total abdominal hysterectomy and bilateral salpingo-oophorectomy with omental biopsy and pelvic lymph node dissection was also performed. Grossly, the left ovary contained a well-defined tan, homogeneous nodule that extended from the ovarian surface into the parenchyma. It measured 1.7 cm in greatest dimension. The distal portion of the left fallopian tube was adherent to this ovarian nodule. Within the omental tissue, a 6-cm well-defined tan, haemorrhagic nodule was noted. The uterus showed several intramural leiomyomas. The pelvic lymph nodes were of normal size and shape.

Microscopically, the left ovary showed a high-grade adenocarcinoma composed of sheets of tumour cells with a predominantly solid pattern and focal glandular differentiation (Figure 2). The sheets of tumour cells were surrounded by a lymphoplasmacytic infiltrate. The tumour cells were pleomorphic with prominent nucleoli. Mitotic figures were easily identified.

The omental mass showed a different histologic picture with a papillary growth pattern (Figure 3), a lack of a lymphoplasmacytic infiltrate and numerous psammoma bodies diagnostic of a papillary serous adenocarcinoma. While the breast and ovarian masses appeared histologically similar and quite different from the omental mass, the immunohistochemical pattern of the ovarian mass and omental mass were similar, showing positivity for oestrogen receptor, p53, CA125 and negativity for progesterone receptor. The breast tissue was not stained for CA125 but was negative for p53 and positive for progesterone receptor.

In addition to patient 1, patient 2 had a synchronous presentation that caused some diagnostic problems: she was initially treated with tamoxifen in view of an oestrogen-receptor-positive breast cancer but had progression of disease with peritoneal carcinomatosis from her ovarian cancer. This showed some response to carboplatin + paclitaxel and subsequently to the pegylated lipsomal doxorubicin prior to the development of recurrent ascites that led to her eventual death. In patient 3, the course was that of a fairly typical platinum-sensitive ovarian cancer that eventually recurred in the liver and had only transient responses. Patient 4 did not demonstrate diagnostic dilemmas, was treated for her subsequent development of ovarian cancer and has not developed metastases at this point. Patients 5, 6 and 8 manifested ovarian cancers at various times after the diagnosis of breast cancer. Peritoneal carcinomatosis predominated in all three, but two developed unusual sites of metastases from ovarian cancer such as brain, bone and lymph nodes as well as abnormalities in CA27.29 that behaved somewhat discordantly with CA125. Patient 6 was not tested, but her family history included breast cancer in several members of her family and a fatal pancreatic cancer in her son, making the mutation likely. The pathologic diagnosis of ovarian cancer in patient 8 was histologically quite similar to her triple negative breast cancer; she responded initially to platinum-based chemotherapy and progressive disease in the peritoneum predominated and eventually led to her death. Although she herself had not been tested for germ-line mutations, her two daughters have tested positive for muBRCA1.

## Discussion

The histological findings in the patient reported in full (and also other findings in several other patients listed) have raised the intriguing possibility that the ovarian cancer diagnosis represents metastases from the breast cancer. Some of the clinical features suggest that both diagnoses may at times co-exist. Perhaps these breast cancer cells lay dormant until a new ovarian cancer development changes the milieu of the surrounding areas and sparks proliferation.

The presence of BRCA mutations may make ovarian cancers more amenable to treatment with DNA damaging drugs such as platinums. Whether this also pertains to breast and pancreatic cancers arising in a BRCA mutated background has not been well studied but has been proposed. Awareness of issues in the diagnosis of these patients’ diseases may enable clinicians to formulate appropriate therapeutic plans, taking into account the possibility of some diagnostic ambiguities as well as the importance of establishing the presence of BRCA mutations. As more cases are studied, differences may emerge between tumours arising in a BRCA1 versus BRCA2 mutation background.

In presenting the range of findings in these patients, we conclude that a coincidental co-existence of breast and ovarian cancers is likely to account for clinical findings. Breast cancer being most common and more likely to appear at an early age, usually precedes the ovarian cancer, but in two instances (both BRCA2 mutation carriers) these cancers occurred synchronously, leading to diagnostic challenges. Even in metachronous presentations, however, overlapping histological findings, unusual site of metastases for ovarian cancer, such as brain and bone, and co-expression of epithelial markers CA125 and CA27.29 may add to the diagnostic dilemma. The most important implication for treatment, however, may lie in establishing the presence of the mutation and the enhanced susceptibility of these tumours to DNA damaging agents.

## Figures and Tables

**Table 1:**
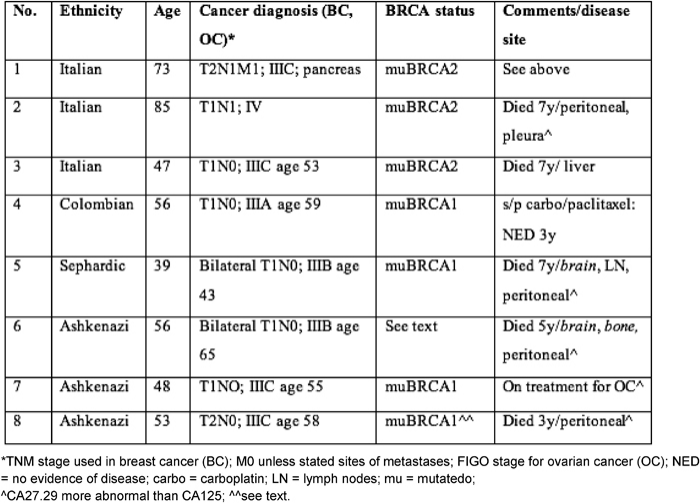
Characteristics of sample patients with both breast and ovarian cancer diagnoses
